# Supplementary material for: Comparison of antibody responses to SARS-CoV-2 variants in Australian children
Source: Nat Commun. 2022 Nov 23;13:7185. doi: 10.1038/s41467-022-34983-2 (PMC9700848; doi:10.1038/s41467-022-34983-2)
Supplement: Supplementary file 1 — Supplementary Information [file 41467_2022_34983_MOESM1_ESM.pdf]

Supplementary Data

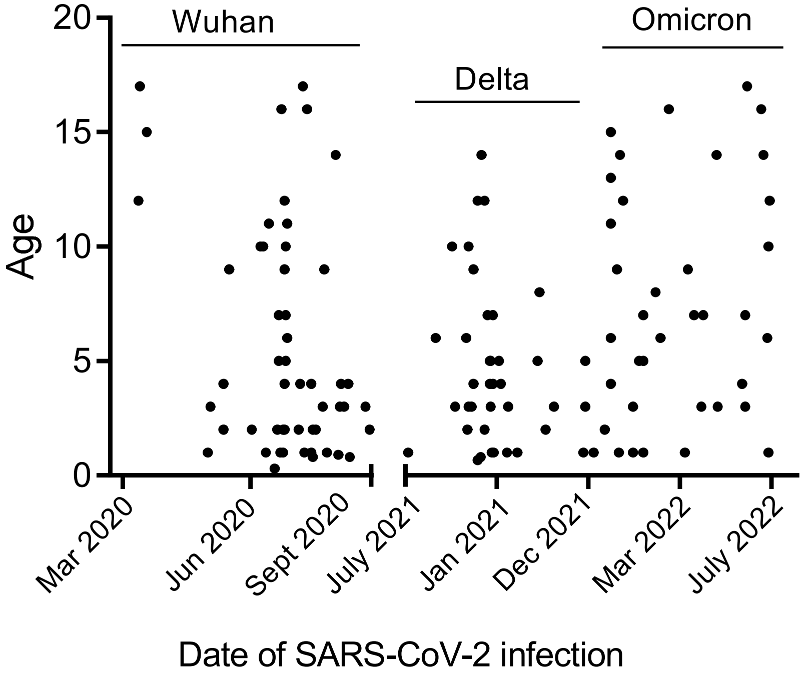

Supp. Figure 1: Overview of the cohort by age and their date of SARS-CoV-2 infection.

Each dot represents a child. N=131.

Supp. Table 1: Baseline characteristics of children included in the analysis

Waves	Wuhan (May 2020-Aug 2020)	Delta (July 2021-Dec 2021)	Omicron (Jan 2022 onwards)	Vaccinated plus Omicron
Number of participants	56	35	16	24
Age, median (IQR), y	4 (2-9)	4 (2-7)	3 (1-3)	10 (7-14)
Sex, female, N (%)	21 (37.5%)	17 (48.6%)	7 (43.8%)	12 (50.0%)
Median time post-PCR/RAT diagnosis, days (IQR)	40 (31-48)	34 (31-40)	31 (23-37)	31 (28-43)
Number of participants with breakthrough infection following each vaccine dose (N) (Median time of infection since last vaccine dose)	-	-	-	
1				N=7 (23 days)
2				N=15 (106 days)
Booster				N=2 (87 days)

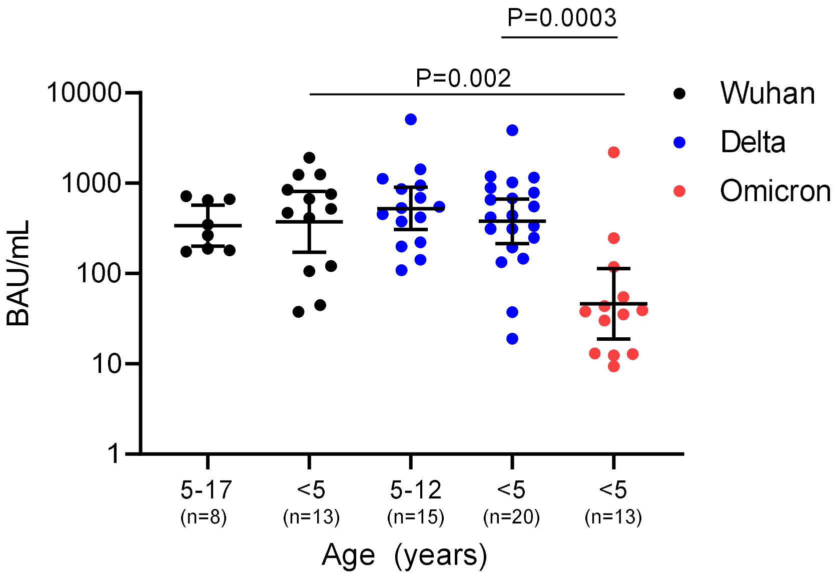

Supp. Figure 2: SARS-CoV-2 IgG **antibody responses in children infected with Wuhan, Delta or Omicron strain stratified by age** as measured by ELISA using the Wuhan S1 antigen.. Each data point represents an individual participant. Data presented as geometric mean concentrations ± 95% confidence intervals. Comparison of S1-specific IgG antibody concentrations were done using a two-sided Mann-Whitney U test. BAU, binding antibody units.

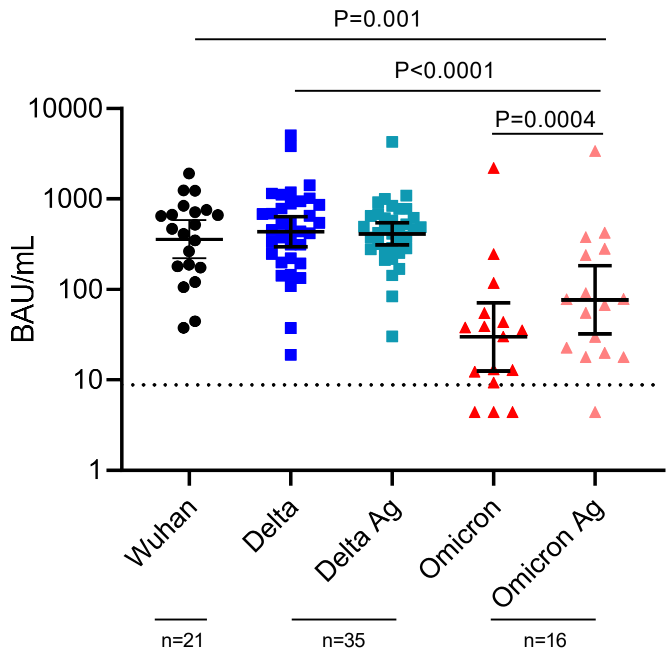

Supp. Figure 3: SARS-CoV-2 IgG responses in children infected with Wuhan (black), Delta (blue) or Omicron (red) strain as measured by ELISA using Wuhan or variant-specific S1 antigen (Ag). Dotted lines indicate seropositivity cut-off. Each data point represents an individual participant. Data presented as geometric mean concentrations ± 95% confidence intervals. Comparison of the S1-specific IgG antibody concentrations between children from different SARS-CoV-2 waves and between variant-specific antibody responses were compared using two-sided Mann-Whitney U test and Wilcoxon-signed rank test, respectively. BAU, binding antibody units.

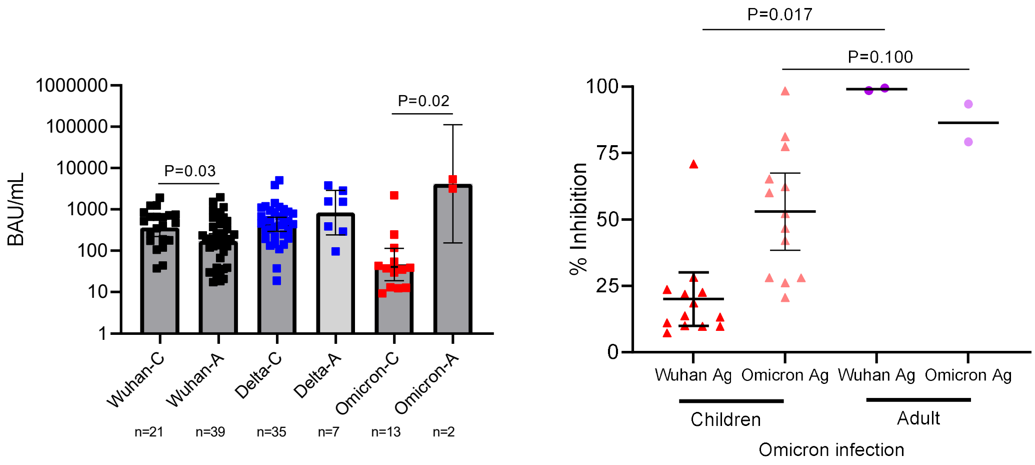

Supp. Figure 4: Comparison of SARS-CoV-2 IgG antibody responses between children and adults. (A) SARS-CoV-2 IgG concentration in children (C) or adults (A) infected with Wuhan (black), Delta (blue) or Omicron (red) strain as measured by ELISA using the Wuhan S1 antigen. (B) Neutralising antibodies of unvaccinated children (red, n=13) or adults (purple, n=2) infected with Omicron measured by SARS-CoV-2 surrogate virus neutralisation assay using Wuhan or Omicron RBD antigen (Ag). Each data point represents an individual participant. Dotted lines indicate seropositivity cut-off. Data presented as geometric mean concentrations ± 95% confidence intervals. Comparison of the S1-specific IgG antibody concentrations from different SARS-CoV-2 waves and variant-specific neutralising antibody responses between children and adults were compared using a two-sided Mann-Whitney U test. BAU, binding antibody units.
