# Supplementary material for: Comparison of antibody responses to SARS-CoV-2 variants in Australian children
Source: Nat Commun. 2022 Nov 23;13:7185. doi: 10.1038/s41467-022-34983-2 (PMC9700848; doi:10.1038/s41467-022-34983-2)
Supplement: Supplementary file 3 — Reporting Summary [file 41467_2022_34983_MOESM3_ESM.pdf]

nature portfolio

Corresponding author(s): Paul Licciardi

Last updated by author(s): Nov 15, 2022

Reporting Summary

Nature Portfolio wishes to improve the reproducibility of the work that we publish. This form provides structure for consistency and transparency in reporting. For further information on Nature Portfolio policies, see our Editorial Policies and the Editorial Policy Checklist.

Statistics

For all statistical analyses, confirm that the following items are present in the figure legend, table legend, main text, or Methods section.

n/a	Confirmed	
☐	☒	The exact sample size (*n*) for each experimental group/condition, given as a discrete number and unit of measurement
☐	☒	A statement on whether measurements were taken from distinct samples or whether the same sample was measured repeatedly
☐	☒	The statistical test(s) used AND whether they are one- or two-sided<br>*Only common tests should be described solely by name; describe more complex techniques in the Methods section.*
☒	☐	A description of all covariates tested
☒	☐	A description of any assumptions or corrections, such as tests of normality and adjustment for multiple comparisons
☐	☒	A full description of the statistical parameters including central tendency (e.g. means) or other basic estimates (e.g. regression coefficient) AND variation (e.g. standard deviation) or associated estimates of uncertainty (e.g. confidence intervals)
☐	☒	For null hypothesis testing, the test statistic (e.g. *F*, *t*, *r*) with confidence intervals, effect sizes, degrees of freedom and *P* value noted<br>*Give P values as exact values whenever suitable.*
☒	☐	For Bayesian analysis, information on the choice of priors and Markov chain Monte Carlo settings
☒	☐	For hierarchical and complex designs, identification of the appropriate level for tests and full reporting of outcomes
☒	☐	Estimates of effect sizes (e.g. Cohen's *d*, Pearson's *r*), indicating how they were calculated

Our web collection on statistics for biologists contains articles on many of the points above.

Software and code

Policy information about availability of computer code

Data collection	No software was used
Data analysis	Prism V9 was used for all analyses

For manuscripts utilizing custom algorithms or software that are central to the research but not yet described in published literature, software must be made available to editors and reviewers. We strongly encourage code deposition in a community repository (e.g. GitHub). See the Nature Portfolio guidelines for submitting code & software for further information.

Data

Policy information about availability of data

All manuscripts must include a data availability statement. This statement should provide the following information, where applicable:

- Accession codes, unique identifiers, or web links for publicly available datasets
- A description of any restrictions on data availability
- For clinical datasets or third party data, please ensure that the statement adheres to our policy

All data generated or analysed during this study are included in this published article (and its supplementary information files). Source data are provided with this manuscript.

Human research participants

Policy information about studies involving human research participants and Sex and Gender in Research.

Reporting on sex and gender	Sex was reported in Supplementary Data Table 1. A total of 131 children were included in this analysis, comprising 57 females (44%). We did not undertake analysis by sex since our sample size for the omicron cohort is small and that previous analyses showed no differences (Toh et al JAMA Netw Open 2022).
Population characteristics	Children <18 years of age were included in this case-ascertained study on the basis of positive PCR or RAT diagnosis for COVID-19.
Recruitment	Suspected SARS-CoV-2 cases and household members of suspected cases were tested by RT-PCR on nasopharyngeal (NP) swabs at The Royal Children's Hospital or by nasal RAT at home. Confirmed SARS-CoV-2 cases and household members were invited to participate. Blood samples were collected approximately one month following PCR/RAT diagnosis. Written informed consent and assent were obtained from adults/parents and children, respectively. Potential biases include inclusion of only those individuals who underwent PCR or RAT testing and may have underestimated the true number of COVID-19 positive participants. However, this is likely to be minimal since most participants were recruited during the earlier Wuhan and Delta waves and the early part of the Omicron wave when testing in Victoria was a high priority.
Ethics oversight	The study was conducted with the approval of the Royal Children's Hospital Human Research Ethics Committee (HREC): HREC/63666/RCHM-2019.

Note that full information on the approval of the study protocol must also be provided in the manuscript.

Field-specific reporting

Please select the one below that is the best fit for your research. If you are not sure, read the appropriate sections before making your selection.

☒ Life sciences ☐ Behavioural & social sciences ☐ Ecological, evolutionary & environmental sciences

For a reference copy of the document with all sections, see nature.com/documents/nr-reporting-summary-flat.pdf

Life sciences study design

All studies must disclose on these points even when the disclosure is negative.

Sample size	As part of this case-ascertained study, children (with or without vaccination) who had a positive COVID-19 diagnosis by PCR/RAT during the Wuhan, Delta and Omicron waves in Melbourne, Victoria were included in this analysis. We included all participants who were confirmed COVID-19 positive during this period and who had available serum samples for analysis. The sample sizes in this study were sufficient for these analyses despite no formal sample size calculations undertaken.
Data exclusions	No data was excluded.
Replication	All biological samples were replicated at least once. All attempts at replication were successful.
Randomization	This was a case-ascertained study, no randomization was done.
Blinding	Investigators were blinded during experimentation and analysis.

Reporting for specific materials, systems and methods

We require information from authors about some types of materials, experimental systems and methods used in many studies. Here, indicate whether each material, system or method listed is relevant to your study. If you are not sure if a list item applies to your research, read the appropriate section before selecting a response.

Materials & experimental systems

n/a	Involved in the study
☐	☒ Antibodies
☒	☐ Eukaryotic cell lines
☒	☐ Palaeontology and archaeology
☒	☐ Animals and other organisms
☒	☐ Clinical data
☒	☐ Dual use research of concern

Methods

n/a	Involved in the study
☒	☐ ChIP-seq
☒	☐ Flow cytometry
☒	☐ MRI-based neuroimaging

Antibodies

Antibodies used

Southern Biotech, Goat Anti-Human IgG-HRP (1:10,000), Cat no: 2040-05 for the SARS-CoV-2 ELISA. Genscript sVNT assay was used.

Validation

Antibodies and reagents used were derived from commercial sources. Validation of the reagents are provided in the respective manufacturer's instructions for use documentation. The SARS-CoV-2 ELISA has been validated on known positive samples and pre-pandemic samples, and has been published previously.
